# Percutaneous Endovascular Aneurysm Repair Complicated by Shallow Femoral Artery Access

**DOI:** 10.7759/cureus.94108

**Published:** 2025-10-08

**Authors:** Fumiya Haba, Motoharu Shimozawa, Kosaku Nishigawa, Shunya Ono, Takeyuki Kanemura

**Affiliations:** 1 Cardiovascular Surgery, IMS Katsushika Heart Center, Tokyo, JPN

**Keywords:** abdominal aortic aneurysm, access failure, percutaneous endovascular aortic repair, shallow common femoral artery, vascular closure device

## Abstract

Percutaneous endovascular aortic aneurysm repair (PEVAR) with vascular closure devices is widely used as an alternative to surgical cutdown. While predictors of access-related complications, such as common femoral artery (CFA) calcification and increased depth, have been reported, excessively shallow common femoral arteries are not well recognized as a risk factor. We report an 82-year-old emaciated male (height 168 cm, weight 43 kg, body mass index 15.2) with a rapidly enlarging abdominal aortic aneurysm who underwent PEVAR using the Perclose ProStyle device (Abbott Vascular, Inc., Redwood City, United States). Despite pre-placement of sutures, hemostasis failed bilaterally due to suture tracts traversing skin and subcutaneous tissue rather than advancing to the arterial wall, necessitating surgical cutdown and additional sutures to achieve hemostasis. Postoperative recovery was uneventful. Computed tomography revealed that both CFAs were located only 3 mm beneath the skin, suggesting that the posterior needle exit may have emerged extracorporeally, entrapping skin and subcutaneous tissue in the suture pathway. This case underscores that extremely superficial CFAs can predispose to failure of percutaneous closure devices. In patients with CFA depth less than 8 mm, operators should exercise caution, avoid excessive upward traction during needle advancement, and consider primary surgical cutdown as a safer alternative.

## Introduction

Percutaneous endovascular aortic aneurysm repair (PEVAR) using vascular closure devices has shown promising results compared to surgical femoral cutdown. The PEVAR trial demonstrated non-inferiority of Perclose ProGlide (Abbott Vascular, Inc., Redwood City, United States) compared to surgical cutdown, with shorter time to hemostasis and procedure completion [1]. Multiple studies have demonstrated high technical success rates of 92-96% for PEVAR [2-4]. Several factors are associated with access failure and complications. These include severe vessel calcification [5,6], decreased renal function, increased common femoral artery (CFA) depth [6], female gender, and advanced age [5]. However, shallow femoral arteries are not included.

We report a case of PEVAR surgery in an 82-year-old male who developed hemorrhage despite the use of a percutaneous vascular closure device for a shallow CFA.

## Case presentation

**Figure 1:**
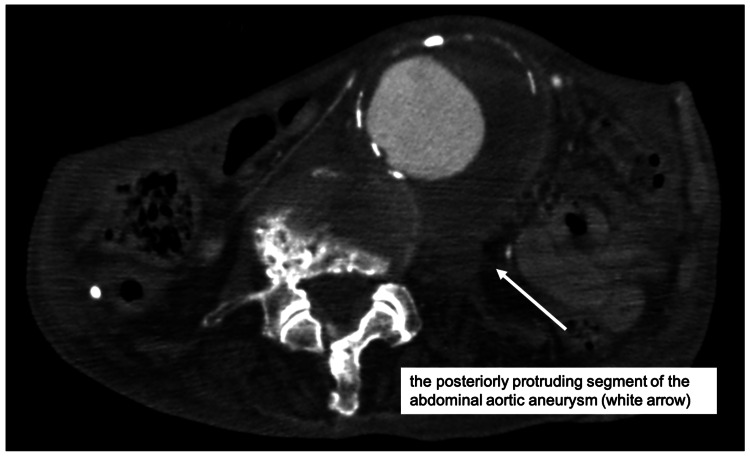
Computed tomography angiography showed an abdominal aortic aneurysm that was enlarged and protruding posteriorly.

**Figure 2:**
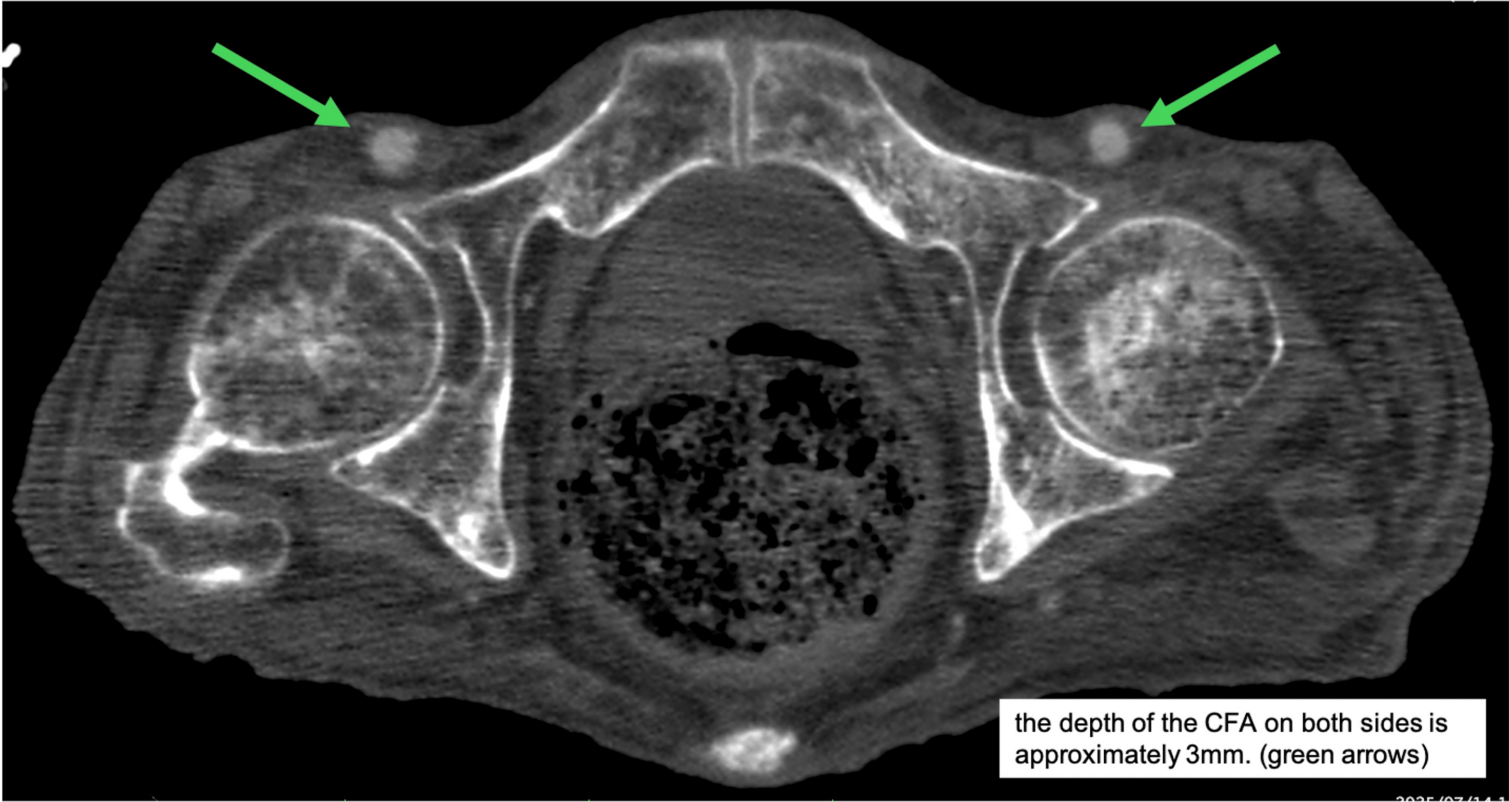
The contrast-enhanced CT scan shows that both CFAs are very shallow.

An 82-year-old man was advised by his primary care physician during a home visit to seek early evaluation at our hospital after progressive enlargement of an abdominal aortic aneurysm was noted. The patient presented five days later. Contrast-enhanced computed tomography demonstrated an abdominal aortic aneurysm measuring 55 mm in diameter, with a rapid increase of 14 mm over the preceding six months. The aneurysm was dorsally protruding and was determined to be at high risk of rupture (Figure 1). The anterior wall of the CFA was located approximately 3 mm beneath the skin bilaterally (Figure 2). The patient was admitted, and surgical intervention was planned.

Under general anesthesia, bilateral CFAs were punctured under ultrasound guidance. Using the Perclose ProStyle device (Abbott Vascular, Inc., Redwood City, United States), two sutures were pre-placed in each artery to facilitate hemostasis at the 8-Fr sheath insertion sites. Through the left CFA, an 18-Fr DrySeal sheath (W.L. Gore & Associates, Flagstaff, AZ, United States) was advanced over an Amplatz Super Stiff guidewire (Boston Scientific Corporation, Marlborough, MA, USA). The main body of the EXCLUDER stent graft (W.L. Gore & Associates, Flagstaff, AZ, United States) was delivered through the DrySeal sheath and deployed immediately below the left renal artery. From the right femoral access, a 5-Fr catheter was advanced into the contralateral gate, and the limb device of the EXCLUDER stent graft was deployed, extending from the main body to the right common iliac artery. Subsequently, the limb device of the EXCLUDER stent graft was advanced and deployed from the main body into the left common iliac artery. Balloon dilation was performed at the graft overlaps, and final angiography demonstrated that there was neither an endoleak nor a migration of the stent graft.

**Figure 3:**
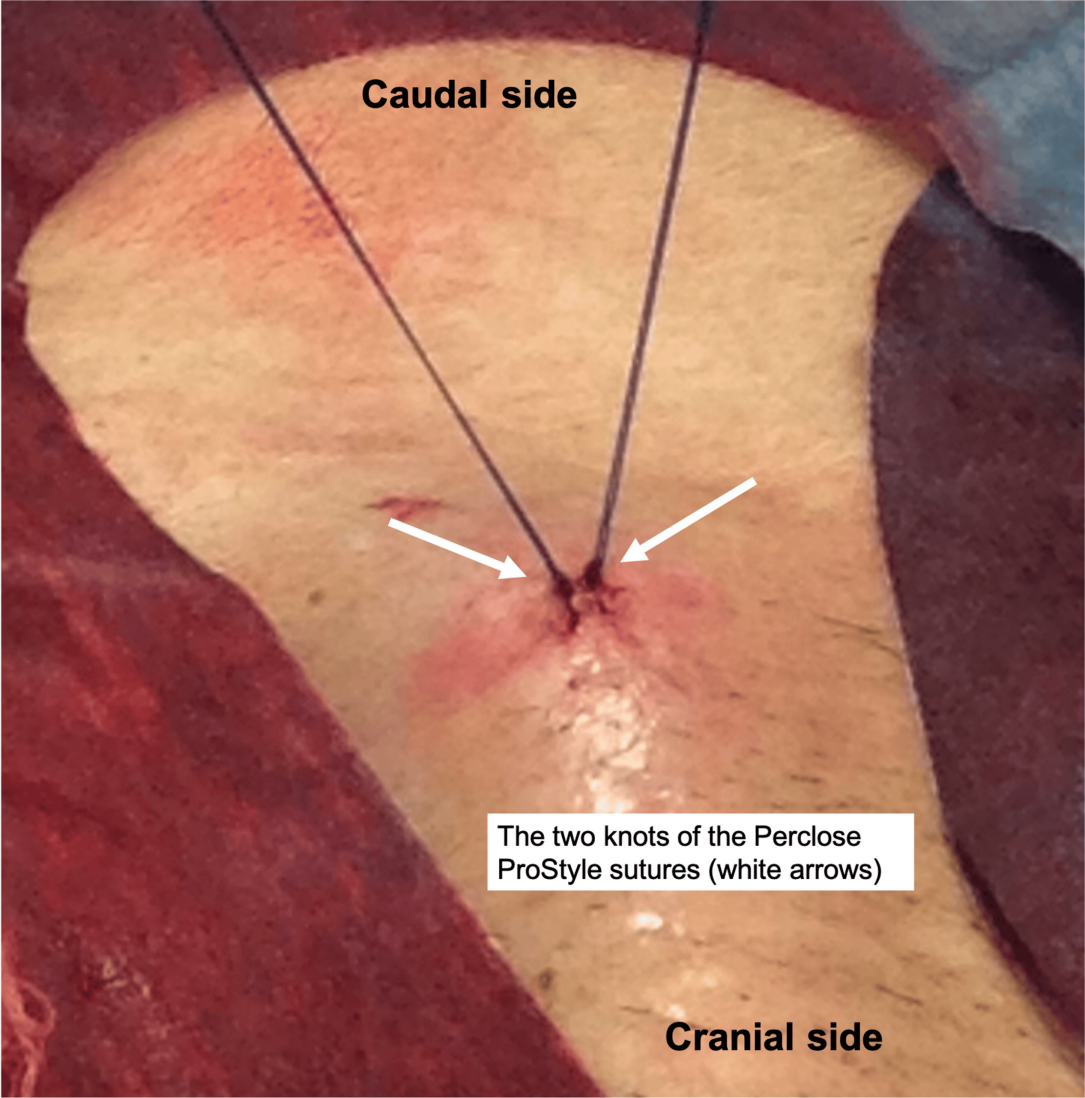
Both knots of the Perclose ProStyle sutures remained external.

Upon removal of the sheaths, the Perclose ProStyle sutures were tightened. However, both sides demonstrated suture tracks traversing the skin and subcutaneous tissue without adequate advancement to the arterial wall, making complete hemostasis impossible (Figure 3). Therefore, surgical cut-down of both CFAs was performed. The puncture sites were exposed, and additional figure-of-eight and purse-string sutures were placed to secure hemostasis. The Perclose ProStyle sutures were removed, and the wounds were closed in layers. Peripheral pulses (dorsalis pedis and posterior tibial arteries) were well palpable bilaterally at the conclusion of the procedure.

The patient’s postoperative course was uneventful. Follow-up contrast-enhanced CT demonstrated no evidence of endoleaks or further aneurysmal enlargement. He was discharged home on postoperative day 4 in good condition.

## Discussion

We describe a case of inadequate hemostasis following PEVAR with the use of a percutaneous vascular closure device in an 82-year-old patient with an abdominal aortic aneurysm. The failure of hemostasis was attributed to the passage of the suture through the skin and subcutaneous tissue, which prevented the knot from advancing to the wall of the CFA.

**Figure 4:**
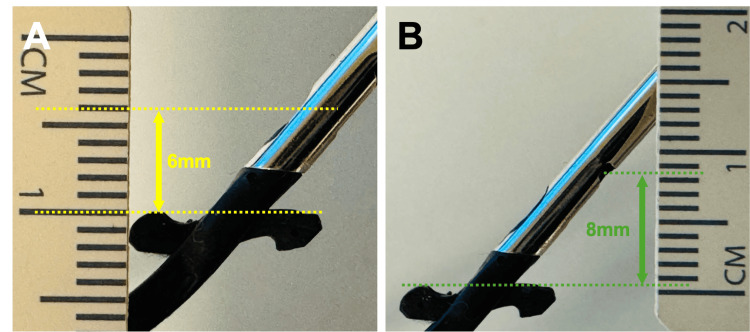
Vertical distance from the anterior surface of the device foot to the needle exit site. (A) The vertical distance between the anterior needle exit site and the device foot is approximately 6 mm. (B) The vertical distance between the posterior needle exit site and the device foot is approximately 8 mm. These images are original and created by the authors.

**Figure 5:**
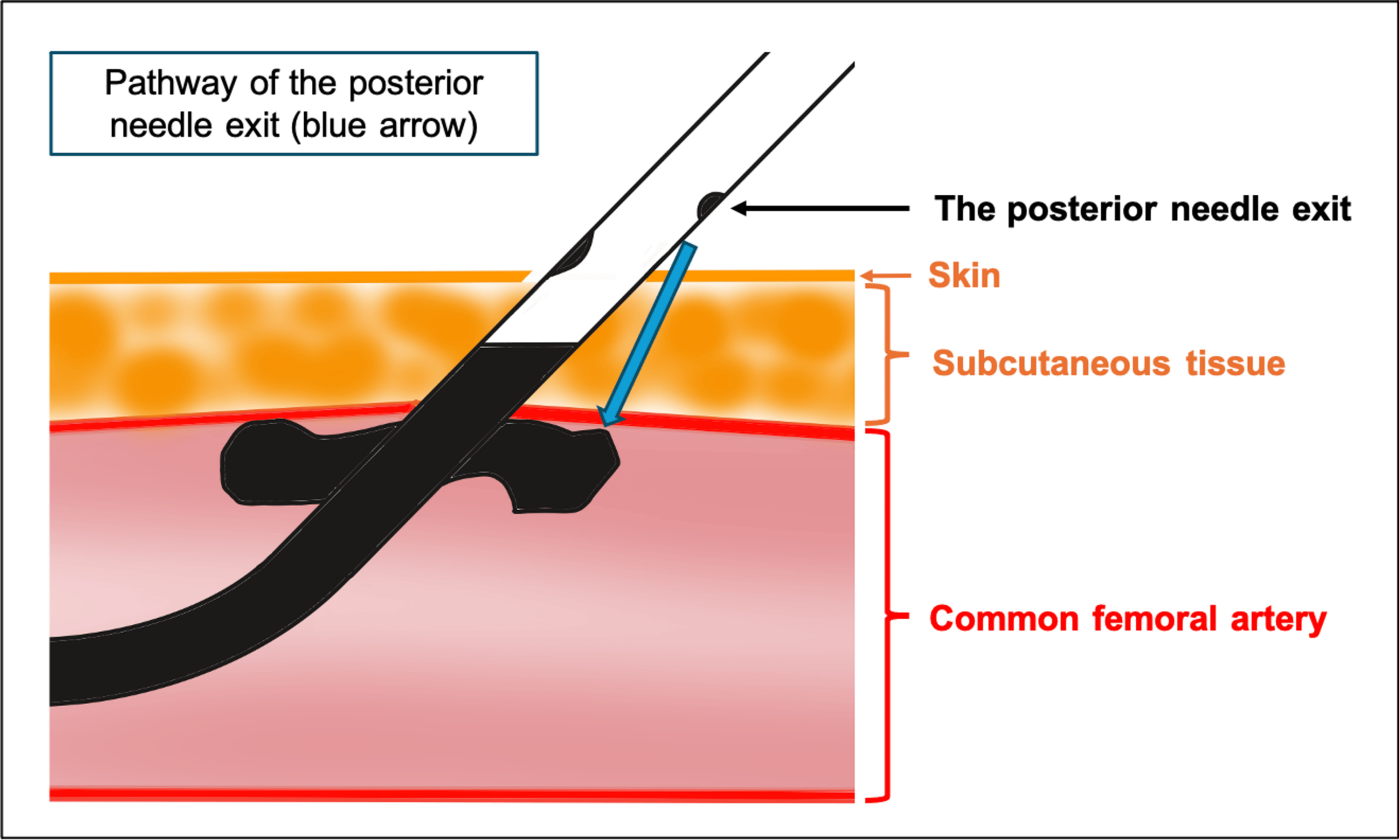
The posterior needle exit site emerged extracorporeally, entrapping skin and subcutaneous tissue between the device foot and the arterial wall. Schematic showing that device retraction led to extracorporeal emergence of the posterior needle exit site and unintended capture of skin and subcutaneous tissue. This figure is original and created by the authors (Figure credits: Fumiya Haba).

The patient was a markedly emaciated male, measuring 168 cm in height, weighing 43 kg, and presenting with a body mass index (BMI) of 15.2. The CFAs were found to be exceptionally superficial, with the anterior wall positioned approximately 3 mm below the skin on both sides. The Perclose ProStyle device functions by deploying its foot against the intimal surface of the CFA, enabling the introducer needles inserted from outside the vessel to pass through cuffs within the foot, thereby creating a continuous suture loop. The distance from the anterior surface of the foot to the needle exit site measures 6 mm anteriorly and 8 mm posteriorly (Figure 4). During needle deployment, the device must be retracted slightly to ensure firm apposition of the foot against the arterial wall, further decreasing the skin-to-artery distance. In this case, it is likely that the posterior needle exit site emerged extracorporeally, allowing skin and subcutaneous tissue to become interposed between the foot and the arterial wall, resulting in inadvertent incorporation of non-vascular tissue into the suture pathway (Figure 5).

According to the Instructions for Use (IFU) of the Perclose ProStyle device, it is noted that when the access vessel is considerably deep, complications such as cuff miss or incomplete knot advancement to the arterial wall may occur, and the operator may need to elevate or compress subcutaneous tissue to obtain backflow. However, the IFU does not include any caution or description regarding extremely shallow CFAs or the risk of device malfunction in such anatomical conditions. Therefore, in patients with very superficial femoral arteries, the operator must rely on anatomical assessment and procedural judgment rather than manufacturer guidance to ensure safe device deployment.

Previous studies have identified both CFA calcification and vessel depth as predictors of access-related complications. Mousa et al. reported that severe circumferential calcification was the most significant independent predictor of conversion to open repair, likely because a rigid, non-compliant arterial wall interferes with proper foot deployment and secure suture engagement [5]. Gradinariu et al. also demonstrated that increased CFA depth was associated with a higher rate of access-site bleeding and closure device failure, suggesting that excessive subcutaneous distance impedes adequate knot advancement [6]. However, while both studies focused on the adverse impact of calcification and greater CFA depth, they did not specifically analyze anatomical situations with very shallow arteries. This case, therefore, suggests that, in contrast to deep or calcified vessels where the knot fails to reach the arterial wall, an overly shallow CFA may cause the posterior needle to exit extracorporeally, leading to suture misplacement and hemostatic failure.

Furthermore, if the suture knot protrudes outside the body due to extracorporeal needle passage, even temporary hemostasis achieved through effective compression may carry a risk of wound infection arising from exposure of the suture material to the external environment. Therefore, superficial placement of the knot should be avoided whenever possible to minimize postoperative infectious complications.

The threshold of 8 mm for CFA depth was proposed based on the structural dimensions of the Perclose ProStyle device: the posterior needle exit site is located approximately 8 mm from the foot surface. Therefore, when the skin-to-artery distance is less than this value, even minimal device retraction can result in the posterior needle emerging outside the vessel wall, as likely occurred in this case. This anatomical consideration provides a mechanistic basis for adopting 8 mm as a critical safety margin below which percutaneous closure may become unreliable.

The PEVAR trial, which was the first multicenter randomized controlled study comparing percutaneous and open femoral access for endovascular aneurysm repair, reported a 6% rate of major access-site complications in the ProGlide group and no procedure-related mortality at 30 days [1]. These findings confirmed the overall safety and efficacy of percutaneous closure in appropriately selected patients. However, the trial excluded patients with “poor CFA quality,” such as circumferential calcification or anatomically unfavorable access, and did not assess extremely shallow arteries. Consequently, while our procedural approach was consistent with current evidence-based practice, this case illustrates that CFA shallowness - unaddressed in both the IFU and the PEVAR trial - may constitute an unrecognized risk factor for device malfunction and hemostatic failure.

To avoid such complications, several technical considerations may be proposed. First, operators should avoid excessive retraction of the device during needle advancement. Second, careful confirmation is required to ensure that the needle exit site has not emerged extracorporeally prior to suture passage. Finally, in profoundly thin patients with extremely superficial CFAs, open surgical cut-down should be considered as a safer alternative to percutaneous closure.

## Conclusions

We reported a case of hemostatic failure following the use of a percutaneous vascular closure device in a patient with shallow CFAs. Based on this experience, we propose that in cases where the depth of the CFA is less than 8 mm, careful attention should be paid to avoid excessive upward traction of the device during needle deployment, and confirmation should be made that the needle exit site does not become externally visible. Furthermore, in patients with extremely superficial femoral arteries, an initial surgical cut-down approach should be considered as a safer alternative to percutaneous closure.
